# Analysis of Policies Based on the Multi-Fuzzy Regression Discontinuity, in Terms of the Number of Deaths in the Coronavirus Epidemic

**DOI:** 10.3390/healthcare9020116

**Published:** 2021-01-22

**Authors:** Xianghui Wang, Chang Chen, Yan Du, Yang Zhang, Chengliang Wu

**Affiliations:** School of Economics and Management, Beijing Forestry University, Beijing 100083, China; wangxianghui@bjfu.edu.cn (X.W.); changchen2020@bjfu.edu.cn (C.C.); duyan2020@bjfu.edu.cn (Y.D.); wuchengliang@bjfu.edu.cn (C.W.)

**Keywords:** coronavirus (COVID-19), policy stringency, regression discontinuity

## Abstract

It has been a year since the outbreak of the coronavirus epidemic 2019 (COVID-19). In the face of the global epidemic, governments in all countries have taken different prevention measures, such as social isolation, mandatory health protection, and the closure of schools and workplaces. The situation of the epidemic has clearly varied from country to country. In this context, research on the impact of policies for the control of the spread of the global epidemic is of great significance. In this paper, we examined data from a sample of 212 countries between 31 December 2019, and 21 May 2020, using multi-fuzzy regression discontinuity. We found that developed countries had relatively low sensitivity to the policy stringency index; however, policy control measures had a significant effect on epidemic control. In addition, the trend analysis showed that the corresponding management and control came into play only after the policy stringency index reached 50 or the policy management reached level II, and the robustness was optimal at this time. Therefore, the governments in all countries should realize that epidemic prevention and control are of great importance. They can strengthen policy stringency to control the spread of the epidemic, considering their national conditions in terms of the economy and health system.

## 1. Introduction

### 1.1. Coronavirus Situation

In December 2019, the coronavirus epidemic broke out in the South China Seafood Market in Wuhan, China, and then quickly swept all over the world. It has now spread to more than 200 countries, and more than 30 million people have been infected, which has also attracted the attention of academic circles. The epidemic has not only affected public health in society but has also had a serious impact on the economy. Specifically, the increase in infected people, the sharp decline in income, the increase in unemployment, and the stagnation of economic development have worried people all over the world [1].

At present, the global coronavirus epidemic is still very severe. The number of infected people has accumulated to 7.24 million as of 26 September 2020, in the United States, the country with the largest number of infections. The United States took highly decentralized measures, and different states had different ways of responding to the epidemic. Eleven states did not strictly close down unnecessary commercial activities, which may be one of the reasons for the rapid development of the epidemic [2]. According to a survey of the American people, nearly half of the people said that they would prioritize reducing the risk of infection and hope to postpone the reopening of non-essential businesses, while the other group (13%), mostly non-partisans with higher income, strongly urged the resumption of work and production [3].

India is currently second to the United States in terms of the number of new infections. The prevention and control policies have not been effective, with a total of 5.9 million people infected there due to poor health and medical conditions, overpopulation, the consequent difficulty in monitoring, and other reasons [4]. The epidemic in Brazil was also not effectively suppressed. Reis analyzed the infection situation in Brazil through a ordinary differential equations (ODE) system model. He believed that the effects of Brazil’s policies on prevention and control were only about half of those of the policies in South Korea and Italy. This may be due to the volatile Brazilian economy. More than 13 million people live in slum areas, which makes it difficult to maintain social distance and good sanitary conditions [5].

The epidemic situation has been relatively controllable in other Asian countries, such as in Singapore and South Korea. In this epidemic, prevention and control measures in Singapore were in place, including the temperature monitoring of foreign tourists implemented in January, strict isolation measures for high-risk areas, and the vigorous tracking of close contacts of sick groups, leading to a total death toll of only 27 [6]. South Korea made use of technology to launch various monitoring and tracking systems and transparently disclosed all tracking data to the public through a central database, effectively controlling the spread of the virus, which indicated that the application of innovative technologies had positive significance for epidemic prevention and control [7].

### 1.2. Research on Effects of Policies for Prevention and Control of the Epidemic

At present, the top priority of research is in the field of medical treatment. However, policies from the government are also indispensable factors for epidemic prevention and control. Policies in response to the epidemic are mainly divided into two types: one is those to reduce morbidity and mortality through various measures, such as confinement policies, social isolation, and public health services; the other is those to implement various fiscal and monetary measures to mitigate the economic loss caused by the epidemic itself and isolation policies [8,9,10,11,12].

This paper focuses on the social epidemic prevention and control policy. In addition to drug treatment, government interventions, such as the shutdown of unnecessary commercial activities and moderate isolation, may help to prevent the spread of infectious diseases [13,14]. Therefore, it is worth studying the prevention and control policies issued by the government. Some scholars have focused on comparing the prevention and control policies of countries. For example, they analyzed the prevention and control policies and results for France, Belgium, and Canada, believing that in the face of a large-scale epidemic, centralized countries would be more effective in allocating resources compared with countries with federal state systems, and it also helps eliminate inequalities among regions [15]. 

Others tested the effectiveness of the policies and made suggestions on policies accordingly. Based on the analysis of the Rio de Janeiro epidemic situation, Crokidakis believed that isolation policy had a significant effect on the prevention and control of the epidemic. Using the susceptible infectives quarantined removed (SIQR) model to calculate, researchers found that the predicted number of infected people after effective isolation was 600 times lower than that with no isolation at all [16]. Sardar’s research on India also proved that isolation policy was effective [17]. Lam determined that border control measures, such as entry restrictions and compulsory quarantine, effectively reduced the number of imported cases, using statistics on the number of infected people. Strict domestic isolation measures, personal hygiene awareness, and community participation were also of great positive significance in preventing the spread of the epidemic [18].

This study provides a new perspective for policy assessment by having conducted an accurate and generalized data analysis based on 18,862 samples with a focus on the influences of policy control on the number of deaths from COVID-19. We propose effective prevention and control policies based on the regression results, which is of great practical significance considering that the epidemic has not yet ended in many countries or regions. The main contributions and innovations of this paper lie in the following points. 

First, the current literature studying epidemic prevention and control policies has mostly used data analysis to prove the effectiveness of policies in a single country or qualitative comparisons of policies in several countries, while data from 212 countries were used in this paper, expanding the research samples and examining the stringency of policies worldwide. Secondly, the multi-fuzzy regression discontinuity model was used, where multiple continuous variables exist, among which a threshold was set in this paper. In this way, individuals on one side of the threshold receive the policy intervention while those on the other side do not, constituting a quasi-experiment near the threshold. 

Since the variable is originally continuous, the differences in output between individuals on these two sides are caused by the intervention. Thirdly, regression discontinuity is rarely used in existing research to study the effects of policies for epidemic prevention and control, largely because the policy evaluation criteria are not clear and the recognition is not accurate. We establish an evaluation model for policy stringency in this paper, which further improves the precision of policy descriptions and provides a more suitable analytical basis for using multi-fuzzy regression discontinuity.

## 2. Materials and Methods

### 2.1. Dataset

The data in this paper were from OurWorldInData, mostly supported by the officers at Oxford University. As one of the largest scientific and accessible publications, OurWorldInData can be accessed free of charge. In 2020, OurWorldInData became one of the main organizations that publish global data and research on coronavirus (COVID-19).

Considering the many factors influencing the coronavirus epidemic, our study finally selected 11 research variables to make the research more accurate, namely, location, date, total number of deaths, policy stringency index, median age, domestic production per capita, total value, extreme poverty, female smokers, male smokers, hand-washing facilities, and the number of hospital beds per 1000 people. People in the world were called on to unite in the fight against the COVID-19 pandemic in the 73rd World Health Assembly from 18 May 2020, to 19 May 2020. The resolution was co-sponsored by more than 130 countries and was adopted unanimously. This was the resolution that the largest number of countries have ever sponsored. 

**Figure 1:**
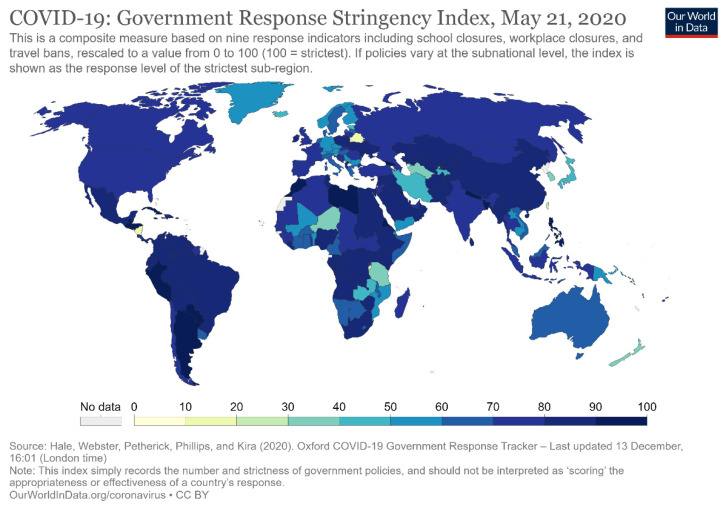
Government response stringency index on 21 May 2020.

Since then, the World Health Organization (WHO) also signed a new agreement with the United Nations High Commissioner for Refugees (UNHCR) on 21 May 2020, to contribute to protecting some 70 million people forcibly displaced due to the effects of COVID-19. This indicates that worldwide attention to the COVID-19 pandemic had risen to an unprecedented level by late May 2020. This can also be seen in the policy stringency index of each country, as shown in Figure 1. The global policy stringency index map on 21 May 2020, showed that most countries in the remaining continents had raised their policy stringency index to 70 or higher, except for some countries in Greenland, Africa, and Central Asia, where the policy stringency indices were around 50. 

This indicated that the policy control of countries around the world had gone through the complete process ranging from early warning, to slight restriction, to strict control by around 21 May 2020. Some countries started to lower the policy stringency index during the second half of 2020, as COVID-19 was gradually stabilizing. Therefore, this study selected the time from 31 December 2019, to 21 May 2020, which basically covered all the important stages of policy control in the whole process of the epidemic, a more complete process of government emergency responses.

The data were from 31 December 2019, to 21 May 2020, generally covering all the important stages of the spread of the epidemic, and the data included 212 countries and regions, covering most regions of the world, which is highly representative. As panel data, after ignoring missing values, the number of observations in the sample reached 18,862. Finally, the variables in this paper and their descriptions are shown in Table 1.

The main research variables included dependent variables (tdeaths_pm), categorical variables (stringency), processing variables (whether the stringency reached 25, 50, or 75), and control variables that represented national or regional characteristics. The specific content is described as follows:

1. Dependent variables: tdeaths_pm refers to the total number of deaths caused by COVID-19 per million people.
2. Categorical variables: Stringency is a comprehensive measurement based on thirteen response indicators, including specific content, such as school closures, workplace closures, and travel bans, mapped to continuous data from 0 to 100 (100 = the most stringent strength), representing the strength of policy control in digital form. Specifically, the Stringency Index is used to measure the difference in government response. As a comprehensive indicator, it can be simply explained as a sum of scores, consisting of nine indicators, using stepped scoring, ranging from 0 to 100. The score is updated every day. The higher the score, the stricter the government’s response (that is to say, 100 = the most stringent response). The score only calculates the stringency of the corresponding government policy but does not indicate whether the corresponding response measures in a county or region are effective or not. This means that for higher scores, the country’s response measures must be better than those with lower scores. The specific calculation method and score coding are shown in Table 2:

Processing variable: Whether the stringency reached 25, 50, or 75, it was recorded as $D_{i}$. Stringency was comprehensively evaluated based on nine specific indicators, and the final value interval was mapped from 0 to 100. In addition, combined with the World Health Organization’s influenza pandemic warning, the alerts can be divided into four levels, of which the first level indicates that the new virus has appeared in animals but with no human infections, the second level indicates that the new virus has begun to infect humans, the third level indicates that the new virus has spread from person to person, and the fourth level indicates that the new virus has begun to spread between countries. 

Of the 13 calculation indicators that constitute the policy stringency index, except for the influenza warning from the World Health Organization, most of their optional scores are at the 3–5 level. Therefore, the policy stringency index can have a standard of four levels. In this way, comparisons among countries and regions can be made through the continuous values of the policy score. An evaluation on policy stringency can also be conducted through the discrete intervals.

Based on this, this paper performed a similar division of the stringency interval, as shown in Table 3:

The regression discontinuity design in Table 2 shows that the stringency had continuous data, but this jumped at the threshold of each level. Specifically, when the stringency was less than or equal to 25, the policy control status of the country or region was loose, and when the stringency was more than or equal to 25, the policy control status of the country or region was relatively loose. If a value near the threshold continues to increase, this will also strengthen the policy control. Generally speaking, government agencies will formulate government control measures based on the stringency of the policy index rather than the specific stringency value. The regression discontinuity was designed as follows based on the above. 

When the stringency value is near 25, 50, or 75, the slightest change will cause a change in the level of policy control, which is likely to cause discrete changes in the number of deaths due to COVID-19. Therefore, the points where the stringency value reached 25, 50, and 75 were regarded as discontinuity points. If the stringency value exceeded these points, it was the treatment group. Otherwise, it was the control group. The treatment effect represented the impact of the change in the policy control level at each point on tdeaths_pm (the total number of deaths per million people). It could be considered that the samples were characterized by a random distribution near each point value, which means that the other characteristics of these samples were the same, or at least, there was no obvious difference. This is also a major advantage of the regression discontinuity design. In this way, missing variables or some of the control variables were solved in the empirical process.

Control variables: Among the factors affecting the deaths in the epidemic, aging, economic development, and sanitary conditions also played essential roles. There was a higher risk of death from COVID-19 in countries or regions with higher proportions of aging populations. There was a relatively lower risk in countries with developed economies and better health and medical conditions where their medical and rescue systems were relatively complete. Considering what is mentioned above, it is necessary to control the above factors to eliminate the differences in objective conditions among countries or regions. To some degree, the avoidance of model setting errors caused by endogenous problems and fewer deviations of regression discontinuity contributed to more accurate effects of policy influences.

Based on this, this study selected some variables for control, mainly including age structure status, economic development, and sanitary facility conditions. Among them, the age structure was represented by the median age that the United Nations predicted according to the ages in each country in 2020. The economic development status included the per capita GDP (2011 constant international dollars as the unit) and presence of extreme poverty (based on the proportion of extremely poor people in each region). The sanitation facility conditions included female smokers (based on the proportion of women who smoke in the total population of the region), male smokers (based on the proportion of men who smoke in the region in the total population), hand-washing facilities (the proportion of the population with basic hand-washing facilities to the total population of the area), and the number of hospital beds per 1000 people (from 2010 to 2020).

**Figure 2:**
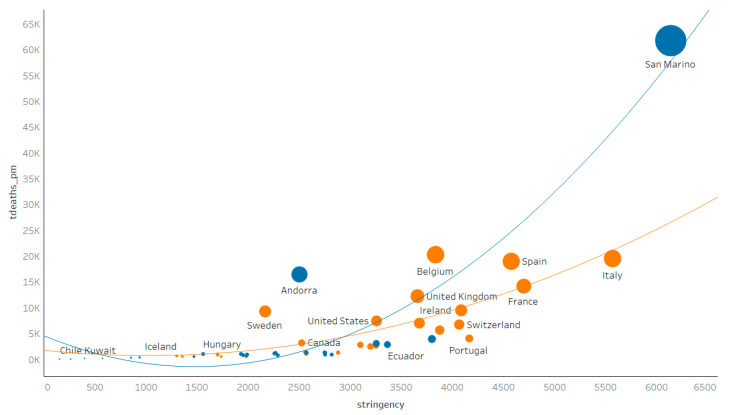
Scatter plot of policy stringency index and tdeaths_pm.

As for the dependent variables and categorical variables mentioned in this paper, Figure 1 was obtained from the samples selected in this study. The tdeaths_pm and stringency generally showed a positive correlation; however, the corresponding correlation coefficient was small. Specifically, some developing countries and developed countries with high mortality were selected to test fit theory. The corresponding fitted line and credible interval are shown in Figure 2. The fitted curve for developing countries was relatively steeper and showed a positive marginal effect, which meant that stringency was more strongly associated with deaths per million people in developing countries (blue dots and lines), while stringency was not sensitive in developed countries (orange dots and lines). Chile and Kuwait are at the bottom left; that is, both the policy stringency index and tdeaths_pm were low, which was in line with the correlation for developing countries.

### 2.2. Model Description

It has been almost one year since the outbreak of COVID-19. In the published literature, simple research models have been used, and the research has focused on the factors affecting the spread of the epidemic. For example, with the help of the epidemic data released by the World Health Organization, Iyanda analyzed the three indicators of age, the proportion of smokers, and out-of-pocket expenditure through spatial analysis [19]. Scholars have also used the same method to analyze and study the infections in certain cities and towns in the United States starting from the counties and confirmed that the relative locations and connectivity of the cities and towns had a greater impact on the spread of the virus within a smaller geographic area [20]. 

Srikanta analyzed the COVID-19 cases in Europe through four spatial regression models, including Geographically Weighted Regression, the Spatial Error Model, the Spatial Lag Model, and Ordinary Least Squares. The results showed that population and income were the key factors affecting the casualties from COVID-19 in European countries [21]. Oztig and Cartenì analyzed the correlation between human mobility and the spread of the epidemic through Negative Binomial Regression and Multiple Linear Regression [22,23]. There are also a small number of scholars who have been committed to establishing a spread model for the epidemic, such as time series models with mixed and normal distributions and a susceptible exposed infected removed (SEIR) spread model suitable for heterogeneous populations to predict the trends of the spread of the epidemic [24,25]. 

However, of all the literature that has studied the factors affecting the epidemic, there have been few studies related to policies from the government. Through linear regression analysis, Sun found that there was no correlation between population density and the spread of COVID-19 under the strict isolation policy in China, indicating that the strict lockdown strategy was very effective in controlling the spread and deterioration of COVID-19 [26]. Chaudhry explored the factors affecting the mortality of those infected with COVID-19 through negative binomial regression analysis. 

The results showed that border blockades and extensive testing had nothing to do with the COVID-19 death rate per million people, but they were significantly conducive to the recovery of the infected, which showed that the government’s prevention and control measures and the speed of the policy had an important impact on the treatment of the infected [27]. In addition, some scholars have analyzed the impacts of the lockdown policies on the epidemic in China through deep learning models and transmission dynamics models [28,29]. Although there are many related documents, the discontinuity regression model has still not been fully applied in the analysis of the impact of policies on the epidemic.

Therefore, the multi-fuzzy regression discontinuity model chosen in this paper, only second to a random experiment, is a method that can avoid the endogeneity problem of parameter estimation. Currently, there are more studies on policy effects employing multi-fuzzy regression discontinuity. However, few are related to epidemiology. A major reason is that regression discontinuity requires a large number of observations near the breakpoints, which is very demanding of data. In addition, it is difficult to compare epidemic policies for infectious diseases that are mostly endemic in small areas, often involving only a single country. The outbreak of COVID-19 provides multiple samples in a short period, together with the spread of the epidemic worldwide and differences in policies in different countries, making it possible to make a comparison and analysis of policies.

In the empirical method of causality analysis, the most optimized is the random experiment. However, as the cost of random experiments is high, other methods need to be adopted. Regression discontinuity is an empirical method that is only second to a random experiment and can effectively analyze the causality between variables by using real constraints.

As shown in Equation (1), the variable ($x_{i}-c$) is the standardized $x_{i}$, and the breakpoint of ($x_{i}-c$) is 0. The interactive term $\gamma \left(x_{i}-c\right)D_{i}$ was introduced to achieve different regression line slopes on either side of the breakpoint. The δ in the ordinary least square (OLS) regression is the estimator of the local average treatment effect (LATE) at $x_{i}=c$. As there is a breakpoint in this regression, it is a regression discontinuity design (RDD).

(1) $$\begin{array}{c} y_{i}=\alpha +\beta \left(x_{i}-c\right)+\delta D_{i}+\gamma \left(x_{i}-c\right)D_{i}+\varepsilon_{i}\left(i=1,\cdots ,n\right), \end{array}$$

The methods for estimating regression discontinuity can mainly be divided into two categories: Sharp RD (SRD) and Fuzzy RD (FRD). The common feature is that the observed individuals were divided on both sides of the discontinuity, which is called the processing state variable. The groups are called the processing group and the observation group. When the categorical variables of the observed individual are higher than a certain threshold or equal to it, the individual enters the processing group. The main difference between the SRD and FRD is as follows: when the processing state variable is a function with definiteness and discontinuity for the driver variable, it is estimated using an SRD (such as Equation (2)). On the contrary, if the driver variable is definite, the probability or expected value of the processing state variable is a function of discontinuous change, and an FRD is used for estimation.

(2) $$\begin{array}{c} D_{i}=\{\begin{array}{cc} 1 & \;\mathrm{if}\;x_{i}⩾25\;or\;50\;or\;75\\ 0 & \;\mathrm{if}\;x_{i}<25\;or\;50\;or\;75 \end{array} \end{array}$$

Therefore, due to various reasons, the samples of stringency in the small scale [25−, 25+], [50−, 50+], or [75−, 75+] were randomly divided so that they could be regarded as a standard experiment. Due to the random division, the Local Average Treatment Effect (LATE) could be consistently estimated when the stringency was nearly 25, 50, or 75, which was an important estimation parameter for this study as shown in Equation (3):

(3) $$\left\{ \begin{array}{c} \mathrm{LATE}\text{\_}25\;\equiv E\left(y_{1i}-y_{0i}|x=25\right)=\underset{x↓25}{lim}E\left(y_{1i}|x\right)-\underset{x↑25}{lim}E\left(y_{0i}|x\right)\\ \mathrm{LATE}\text{\_}50\;\equiv E\left(y_{1i}-y_{0i}|x=50\right)=\underset{x↓50}{lim}E\left(y_{1i}|x\right)-\underset{x↑50}{lim}E\left(y_{0i}|x\right)\\ \mathrm{LATE}\text{\_}75\;\equiv E\left(y_{1i}-y_{0i}|x=75\right)=\underset{x↓75}{lim}E\left(y_{1i}|x\right)-\underset{x↑75}{lim}E\left(y_{0i}|x\right) \end{array}\right.$$

First, we adopted Equation (4) to estimate the regression discontinuity model but encountered a problem in the estimation process. Breakpoint regression is based on a local random experiment, and only observations near the breakpoint can be used; however, the conventional methods use all samples. Therefore, research is needed to limit the value range of the stringency to (stringency−h and stringency+h), where h is the bandwidth. To date, the value has not been determined; therefore, non-parametric regression was chosen to overcome the constraints of the specific function form and select the optimal bandwidth by minimizing the mean square error. 

In non-parametric regression, the kernel regression method was generally adopted. The weight was calculated using the kernel function, and the observation value within the bandwidth h was weighted and averaged. Since the boundary nature of nuclear regression is not good, the focus of this study was on the value of the regression function at the endpoint. Finally, this study adopted local linear regression to estimate the model, while resorting to the kernel function to select the bandwidth to minimize the objective function shown in Equation (4).

(4) $$\begin{array}{c} \underset{\left|\alpha ,\beta ,\delta ,\gamma \right|}{min}\overset{n}{\underset{i=1}{\sum }}\;K\left[\left(x_{i}-c\right)/h\right]{\left[y_{i}-\alpha -\beta \left(x_{i}-c\right)-\delta D_{i}-\gamma \left(x_{i}-c\right)D_{i}\right]}^{2} \end{array}$$

K() is the kernel function. For regression discontinuity, the more popular kernel functions are triangular kernel (K_1) and rectangular kernel (K_2), as shown in Equation (5); z=[c−h,c+h].

(5) $$\begin{array}{c} \{\begin{array}{l} K\_1=1-\left|z\right|)\cdot 1(\left|z\right|<1)\\ K\_2=\frac{1}{2}\cdot 1(\left|z\right|<1) \end{array} \end{array}$$

If a rectangular kernel is adopted, this is equivalent to standard OLS regression, as well as parameter regression. The essence of local linear regression is performing weighted least squares estimation on a small scale, z=[c−h,c+h]. This weight is calculated using the kernel function. The closer the point to c, the greater the weight. The bias and variance of the kernel regression estimator for a large sample are shown in Equation (6), where m() represents the corresponding model.

(6) $$\begin{array}{c} \{\begin{array}{l} Bias\left(c_{0}\right)\equiv E\left[\hat{m}\left(c_{0}\right)\right]-m\left(c_{0}\right)=h^{2}\left[m^{\prime }\left(c_{0}\right)\frac{f^{\prime }\left(c_{0}\right)}{f\left(c_{0}\right)}+\frac{1}{2}m^{\prime \prime }\left(c_{0}\right)\right]\int_{-\infty }^{+\infty }\;z^{2}K\left(z\right)dz\\ Var\left[\hat{m}\left(c_{0}\right)\right]=\frac{1}{nh}\frac{\sigma_{\varepsilon }^{2}}{f\left(c_{0}\right)}\int_{-\infty }^{+\infty }\;K{\left(z\right)}^{2}dz+o\left(1/nh\right) \end{array} \end{array}$$

Specifically, a multi-breakpoint method was adopted to determine the significance of the RDD estimator at multiple discontinuities to avoid false discontinuities. In addition, the fuzzy breakpoint regression adopted in this paper is based on precise breakpoint regression. The difference between the two is that accurate breakpoint regression is at the breakpoint, and the probability of the individual being processed jumps from 0 to 1, while fuzzy breakpoint regression is at the breakpoint, and the probability of the individual being processed jumps from a to b, where 0<a<b<1. In addition, fuzzy breakpoint regression holds that the processing variable D is not completely determined by the grouping variable x. For example, consider studies on the correlation between retirement and household consumption, exploring changes in household consumption before and after retirement. Due to the national policy, residents have a legal retirement age, but it is not guaranteed that everyone will retire in accordance with the provisions; that is, there are early retirement, normal retirement, and delayed retirement. In this case, the fuzzy breakpoint regression method is more applicable.

It is also affected by other factors. Ignoring these factors causes the disturbance term ε to be related to the processing variable D, leading to endogenous problems, which in turn makes the OLS estimates inconsistent. If x is given, $\left(y_{1}-y_{0}\right)$ is independent of D. Based on$\;\left(y_{1i}-y_{0i}\right)⊥D_{i}|x_{i}$, it is assumed that the fuzzy breakpoint regression LATE can be obtained as shown in Equation (7). The numerator is the LATE of the accurate breakpoint regression, and the denominator is the jump (b−a) in the probability of being processed (that is, the propensity score) at breakpoint c. Equation (7) is an extension of the exact breakpoint regression expression. In the case of an exact breakpoint (b−a=1), the expression for the exact breakpoint regression is obtained.

(7) $$\begin{array}{c} LATE\equiv E\left[\left(y_{1}-y_{0}\right)|x=c\right]=\frac{\underset{x↓c}{lim}\left(y|x\right)-\underset{x↑c}{lim}\left(y|x\right)}{\underset{x↓c}{lim}E\left(D|x\right)-\underset{x↑c}{lim}E\left(D|x\right)} \end{array}$$

Since the numerator of Equation (7) is the LATE of the accurate regression discontinuity, the accurate regression discontinuity (local linear regression, etc.) can be used to estimate the numerator. In addition, the denominator and the numerator are almost the same in form and so can also be estimated by the correlation method of accurate regression discontinuity. The only difference is that the result variable y needs to be replaced by the processing variable D.

The instrumental variable method can also be used to complete fuzzy breakpoint regression. Z1=1(x≥c) was defined to determine whether the grouping variable was greater than or equal to the breakpoint. If it is, then Z1 must be related to the processing variable D, which meets the relevance requirements of the instrumental variables. In addition, Z1=1(x≥c) is equivalent to a local random experiment near the breakpoint, and so D is the only one to be relied on to affect the outcome variable. This is not related to the disturbance term, which meets the exogenous requirements of the instrumental variables. Therefore, Z1 can be used as an effective instrument variable for D, and, on this basis, two-stage least square (2SLS) regression can be used for estimation. If the same bandwidth is used, the 2SLS estimator and the local linear regression estimator of the rectangular kernel are numerically the same.

## 3. Results

### 3.1. Robustness Tests

#### 3.1.1. Games–Howell Test

The Games–Howell test is a non-parametric post-analysis method used for multiple comparisons of two or more samples. The Games–Howell test is somewhat similar to Tukey’s post hoc test [30]. However, unlike the Tukey test, it does not assume a homogeneity of variance or equal sample size. Therefore, the Games–Howell test can be applied to situations where the hypothesis of Tukey’s test is not true. The Games–Howell test and Tukey’s test usually produce similar results. It could be assumed that the data had the same variance and the same sample size. The Games–Howell test uses the Welch–Satterthwaite degree of freedom equation, also known as the set degrees of freedom, based on Tukey’s studentized range distribution. It uses the ranks of observations instead of the original sample observations.

The Games–Howell test is defined as

(8) $$\begin{array}{c} {\bar{x}}_{i}-{\bar{x}}_{j}>q_{\sigma ,k,df} \end{array}$$

In the above, σ is the standard error:

(9) $$\begin{array}{c} \sigma =\sqrt{\frac{1}{2}\left(\frac{s_{i}^{2}}{n_{i}}+\frac{s_{j}^{2}}{n_{j}}\right)} \end{array}$$

The calculation of the degrees of freedom adopted Welch’s correction.

(10) $$\begin{array}{c} \frac{{\left(\frac{s_{i}^{2}}{n_{i}}+\frac{s_{j}^{2}}{n_{j}}\right)}^{2}}{\frac{{\left(\begin{array}{c} s_{i}^{2}\\ n_{i} \end{array}\right)}^{2}}{n_{i}-1}+\frac{{\left(\frac{s_{j}^{2}}{n_{j}}\right)}^{2}}{n_{j}-1}} \end{array}$$

Therefore, a confidence interval could be formed.

(11) $$\begin{array}{c} {\bar{x}}_{i}-{\bar{x}}_{j}\pm t\sqrt{\frac{1}{2}\left(\frac{s_{i}^{2}}{n_{i}}+\frac{s_{j}^{2}}{n_{j}}\right)} \end{array}$$

**Figure 3:**
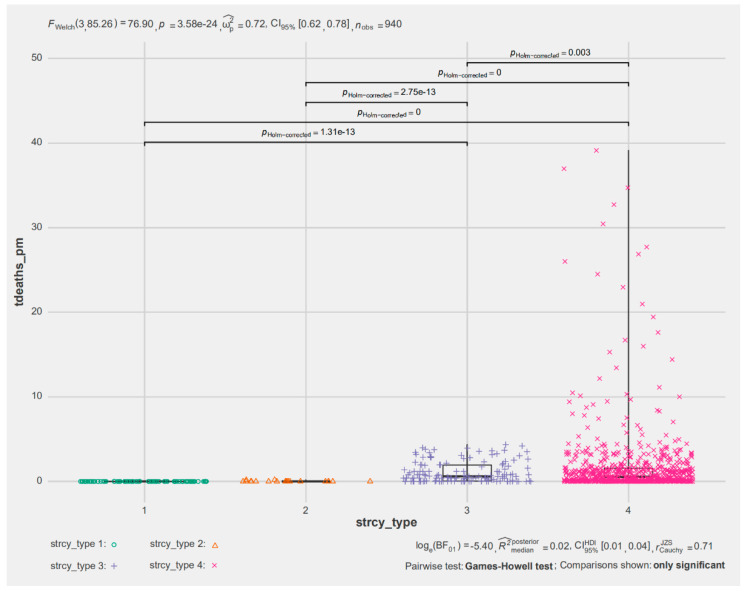
Games–Howell test of deaths per million people under different policy control levels.

As shown in Figure 3, the value of the corresponding F statistic was 76.90 in the Games–Howell test, and the corresponding overall *p* value was less than 0.01, which shows that the overall effect of policy control at the four levels is very significant. Namely, for tdeaths_pm, there are obvious differences in the different policy control levels. Specifically, in the 940 samples, except for the missing values, as the policy control intensifies, the mean and variance of tdeaths_pm continue to increase. However, there was no significant difference between the first and second levels of policy control. The *p* values of the other pairwise comparisons were all no more than 0.002. This means that tdeaths_pm and stringency generally maintain a positive correlation. Namely, the policy control of various countries can be related to the trend of the number of deaths from the epidemic globally.

#### 3.1.2. Continuity Test

In fact, regression discontinuity design implies several important preconditions:

Breakpoint regression has a pre-determined assumption that the conditional density of the control variable is continuous at the corresponding breakpoint. This is because if there is a jump in the conditional density function of the control variable, the corresponding processing effect is missing. As shown in Table 4, when the stringency is 25, the conditional density function with the three variables of m_age, e_poverty, and handwash is not continuous at the breakpoint. When the stringency is 50, the conditional density function of the three variables of gdp_pc, e_poverty, and m_smokers is not continuous at the discontinuity. When the stringency is 75, only the conditional density function of the two variables m_smokers and hbp is continuous at the breakpoint. In conclusion, when the breakpoint is at the stringency of 75, there are relatively obvious gaps in its processing effect.

Another pre-determined hypothesis of regression discontinuity is the following: the jump in the dependent variable at the breakpoint only comes from the processing variable, and there is no significant relationship with the control variable. In fact, in the case of discontinuity 25, the continuity of the drive variable (stringency) is the best. In addition, stringency has only slight jumps on both sides of breakpoint 50, and it could be considered that the processing variables obey a random distribution. At this point, the regression discontinuity design adopted is more reasonable. However, on both sides of the discontinuity 75, the jump in the processing variables is relatively obvious, indicating that the estimation is less robust at this point. 

### 3.2. Multi-Fuzzy Regression Discontinuity 

**Figure 4:**
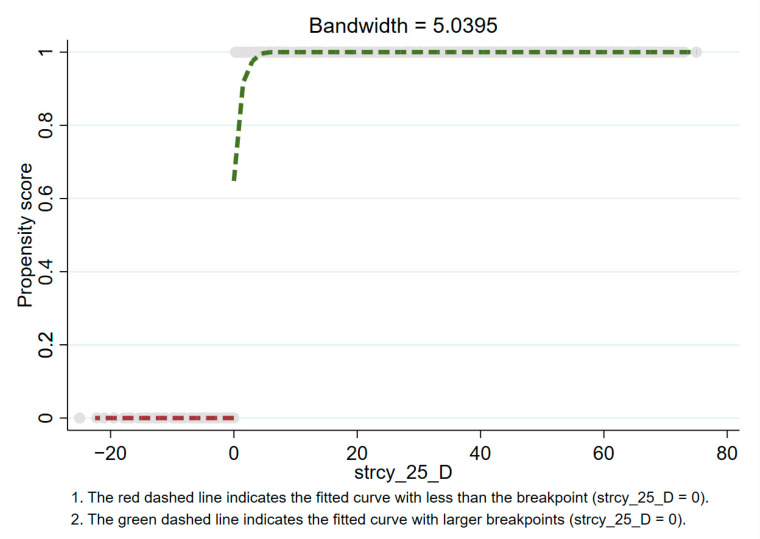
Propensity score at stringency = 25 (based on fuzzy breakpoint regression).

**Figure 5:**
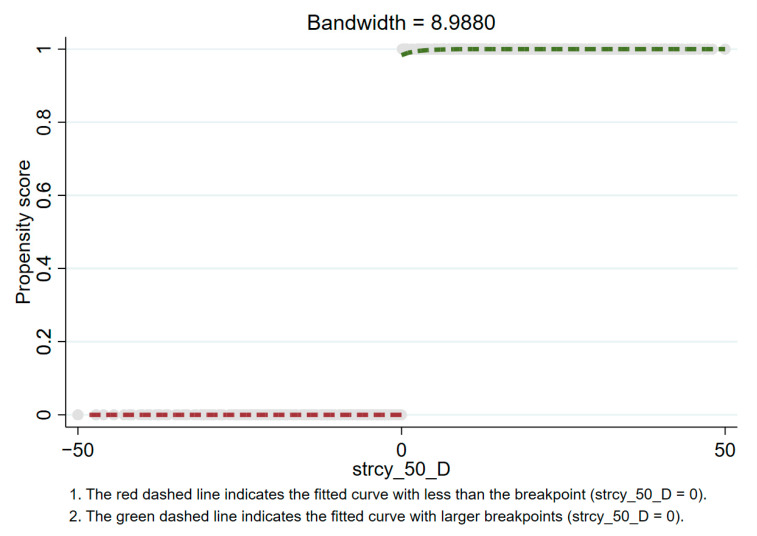
Propensity score at stringency = 50 (based on fuzzy breakpoint regression).

**Figure 6:**
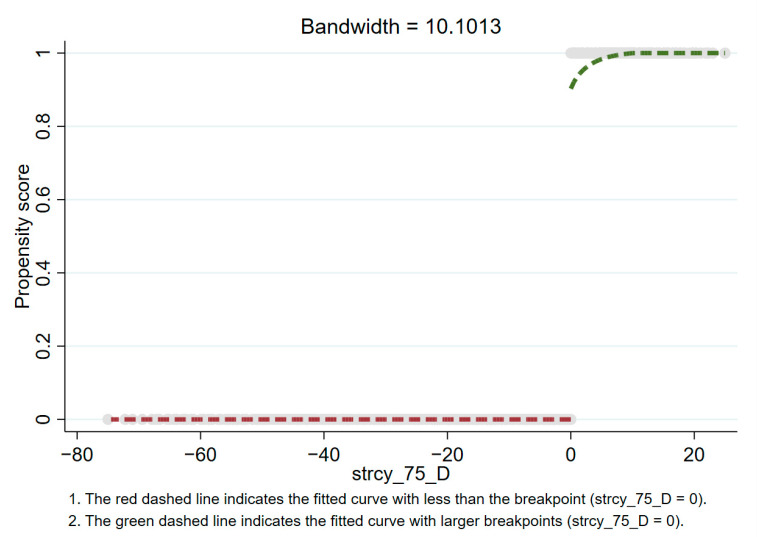
Propensity score at stringency = 75 (based on fuzzy breakpoint regression).

According to Figure 4, Figure 5 and Figure 6, there is an obvious jump when the stringency reaches 25, 50, and 75. This indicates that when the policy stringency index is at these breakpoints, the death toll does show an obvious downward trend. Specifically, the fuzzy regression discontinuity through the optimal bandwidth and triangular kernel is shown in Figure 4 when the stringency is 25. It can be seen that there is a big jump in the propensity score at the breakpoint 25. Before the stringency reaches 25, the propensity score is almost equal to 0. After the stringency reaches 25, the propensity score rises to above 0.6. This means that when the stringency exceeds 25 breakpoints, there is more than a 60% probability that tdeaths_pm will decrease. At the breakpoint of stringency at 50 (as shown in Figure 5), there is an extremely obvious jump in the propensity score. Before the stringency reaches 50, the propensity score is almost equal to 0, and after the stringency reaches 50, the propensity score rises to above 0.9.

This means that when the stringency exceeds 50 breakpoints, there is a more than 90% probability that tdeaths_pm will decrease. Similarly, at the breakpoint of a stringency of 75 (as shown in Figure 6), the propensity score also has a big jump. Before the stringency reaches 75, the propensity score is almost equal to 0. After the stringency reaches 75, the propensity score rises to 0.8 or more. This means that when the stringency exceeds the 75 breakpoint, there is a more than an 80% probability that tdeaths_pm will decrease. In summary, at the breakpoint of the stringency at 50, the effect of fuzzy breakpoint regression is the most obvious. The corresponding policy control measures need to be escalated to the third level of policy control to have the most obvious effect.

According to the statistical test, as shown in Table 5, the *p* value of each statistical indicator is less than 0.05, and the 95% confidence interval does not contain 0 at stringency = 25, 50, and 75, which means that stringency generally controls the tdeaths_pm. Among them, the effect value is the smallest at stringency = 25, only −0.3718, and the effect value is the largest at stringency = 50, which is −34.4109. Therefore, it can be concluded that for multiple discontinuity, an increase in stringency will result in a decrease in the number of deaths per million people, especially when the stringency reaches the third level; this has the strongest inhibitory effect on the number of deaths per million people (LATE = −34.4109).

### 3.3. Bandwidth Sensitivity Test

The estimation process for breakpoint regression requires that the candidate samples be around the discontinuity. If the sample is sufficient, it can completely replace a corresponding random experiment, and then, we can use local linear estimation and calculate according to the different kernel functions. Therefore, the bandwidth selection plays an important role in the results of the regression discontinuity, and the bandwidth selection is actually a trade-off between unbiasedness and effectiveness.

The closer the distance between the data and the discontinuity, the more likely the homogeneity of the sample is to be established, but the available samples will also be wasted; the farther the distance between the data and the discontinuity, the harder the homogeneity of the samples will be to achieve; however, the regression efficiency can be improved. Due to the fact that fewer data are distributed near the discontinuity, the samples on both sides of the discontinuity have difficulty in meeting the requirements. Therefore, discussion can only be carried out in the case of limited samples. The bandwidth scaling combination was chosen to test how robust the estimation results were in this paper.

As shown in Table 6, at the discontinuity stringency of 25, the corresponding average effect is only significant at the optimal bandwidth (the *p* value is 0.0340) but not significant at either 0.5 times (4.5) or 2 times (18). At the stringency of 50, the fuzzy regression discontinuity is very significant at each optimal bandwidth. Finally, at the stringency of 75, the fuzzy regression discontinuity is not significant at 0.5 times the optimal bandwidth (4.5) (the *p* value is 0.5420). On the whole, the fuzzy regression discontinuity of this study is the most robust at the stringency of 50.

**Figure 7:**
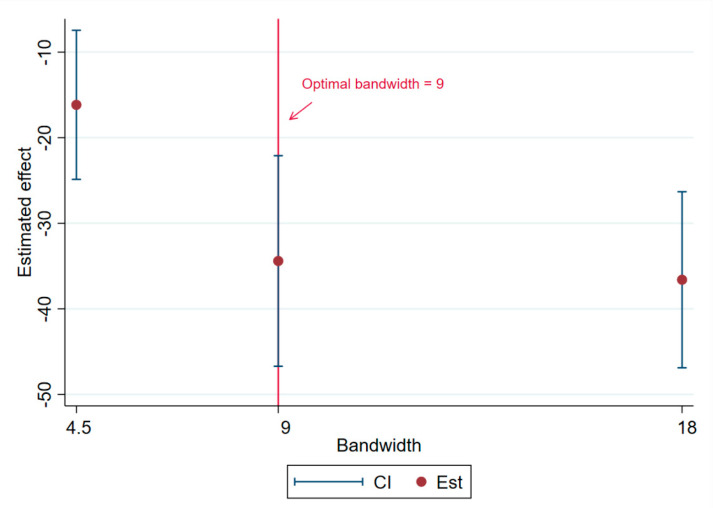
Bandwidth dependence when the fuzzy breakpoint is 50.

Specifically, as shown in Figure 7, when the fuzzy regression discontinuity is at stringency = 50, the dependence of the estimated value on the bandwidth is not obvious. In the case of 0.5 times the optimal bandwidth (4.5), the estimated value is −16.1610 and the confidence interval does not contain 0. The estimated values (as shown by the vertical red line in Figure 7) of the optimal bandwidth (9) and two times optimal bandwidth (18) are −34.4109 and −36.6040, respectively; the corresponding confidence interval is also far less than 0. This means that at a discontinuity of 50, the fuzzy regression discontinuity can strongly prove that policy control has a strong mitigation effect on deaths from COVID-19.

## 4. Discussion and Research Prospects

Epidemic prevention and control are the priority. Although stringent national policies may lead to serious economic recessions or ethical problems, governments should still adhere to policies and regulations that are closely related to epidemic prevention and control. We must recognize that society is a community of life where all human beings live, and only global cooperation in the fight against an epidemic can control its further infections and ensure people’s health. Everyone should comply with national policies with a responsible attitude and isolate themselves from others to ultimately stop the spread of the epidemic.

The assessment of this research is as follows in terms of its strengths and limitations. One strength of this study is that we focused on the impact of policies on the number of deaths from COVID-19, which is different from general epidemiological studies, which focus only on aspects such as drug treatment and infection tracking and prediction. The other strength of this study is that the important role of policy control was proven through the policy stringency index and a powerful tool in casual inference—breakpoint regression.

On the other hand, a limitation of this study is that policy control was measured only by the policy stringency index, and other methods for causal inference applicable to the effect assessment, such as DID (double difference method) and PSM (propensity score matching), were not used in this study. Thus, more methods are needed for robustness testing. Therefore, future studies should perform analysis through multiple causal inference tools. In addition, it is important to find more appropriate indicators of policy control. Finally, there is a lack of empirical studies on local areas (e.g., Asia) and inter-regional comparisons through which key factors influencing the spread of COVID-19 might be identified.

## 5. Conclusions

It can be seen that the epidemic is still severe. More research on the causal relationship between policies and the number of deaths from the epidemic will be helpful for optimizing policies for the prevention and control of the epidemic and stopping the spread of the epidemic.

Based on the above research, the following conclusions can be drawn:

1. Based on visual analysis and the Games–Howell test, we found that the level of policy control in various countries largely matched up with the trend of the number of deaths from the epidemic. Among them, developing countries are more sensitive to policy stringency and perform better in epidemic prevention and control.
2. In the multiple fuzzy regression discontinuity of the policy stringency, the estimated value at each discontinuity is very significant. Taking the triangular core as an example, when the policy stringency is 50, the effect value reaches the maximum value (LATE = −34.4109)—that is, the number of deaths per million people drops by about 34 people when the policy stringency index reaches the third level, and it has the strongest inhibitory effect on the number of deaths from the epidemic at this time.

The correlation between the policy stringency index and the number of deaths from COVID-19 in the world was accurately analyzed to prove the importance of the policies for the prevention and control of the epidemic. Based on the above conclusions, the following recommendations are put forward for effectively mitigating the current world epidemic:

1. Developed countries are less sensitive to policy control; there is an urgent need for relevant governments to strengthen policy control and take continuous and strict social isolation measures. Instead, developing countries are more sensitive to policy control; however, they need to maintain strong policy control sensitivity over time and gradually improve the basic medical systems to cope with sudden outbreaks.
2. In response to the deaths of the epidemic, policy control plays a significant role. Countries should attempt to strengthen policy control. Due to the lag and superposition of policy control effects, epidemic prevention and control will be a longer process. Therefore, more consistent prevention and control procedures must be formulated based on the actual conditions of each country.
3. The government should take measures from multiple aspects. Social isolation should be implemented, as well as comprehensive surveys, resident temperature tracking, and information tracking. Besides, the government should also improve the construction of medical institutions and equipment, and optimize the public health system to achieve both the prevention and control of the epidemic.

## Figures and Tables

**Table 1:** Summary of variable.

Types of Variables	Variables	Definition of Variables	Source
Space	Location	Where it is located	OurWorldInData
Time	Date	When it is observed	OurWorldInData
Dependent variables	tdeaths_pm (total number of deaths per million people)	The total number of deaths from COVID-19 per million people	ECDC (European Centre for Disease Prevention and Control)
Categorical variables	Stringency	Stringency is a comprehensive measurement based on 9 response indicators, including specific content such as school closures, workplace closures and travel bans, mapped to continuous data from 0 to 100 (100 is the most stringent strength), representing the strength of policy control in digital form	OxCGRT, from Blavatnik School of Government
Age structure variables	m_age (median age)	Based on the UN’s predictions on the ages in various countries in 2020	UNPD (United Nations population division) and World population prospects, 2017
Economic development variables	gdp_pc (gross domestic production per capita)	GDP calculated by purchasing power parity (in international dollars at constant prices in 2011)	World Development Indicators from World Bank and Database of World Comparison Program
	e_poverty (extreme poverty)	The proportion of extremely poor people in each region since 2010	World Development Indicators from World Bank and Database of World Comparison Program
	f_smokers (female somkers)	The proportion of female smokers in total people	World development indicators from World Health Organization and database of World Health Observation Station
Sanitation conditions variables	m_smokers (male smokers)	The proportion of male smokers in total people	World development indicators from World Health Organization and database of World Health Observation Station
	handwash	The proportion of people with their own handwashes in their regions	NNSD (United Nations Statistics Division)
	hbp (hospital beds per thousand people)	The hospital beds for every thousand people	OECD (Organization for Economic Cooperation and Development); Eurostat; World Bank and trackers from governments in different countries

**Table 2:** Calculation methodology and score coding of government response stringency index.

Policy Stringency	Levels of Policies for Management and Control
School closures	0—No measures 1—Recommend closing 2—Require closing (only some levels or categories, e.g., just high school or just public schools) 3—Require closing all levels
Workplace closures	0—No measures 1—Recommend closing (or work from home) 2—Require closing (or work from home) for some sectors or categories of workers 3—Require closing (or work from home) all but essential workplaces (e.g., grocery stores and doctors)
Cancel public events	0—No measures 1—Recommend cancelling 2—Require cancelling
Restrictions on gatherings	0—No restrictions 1—Restrictions on very large gatherings (the limit is above 1000 people) 2—Restrictions on gatherings between 100–1000 people 3—Restrictions on gatherings between 10–100 people 4—Restrictions on gatherings of fewer than 10 people
Close public transport	0—No measures 1—Recommend closing (or significantly reduce volume/route/means of transport available) 2—Require closing (or prohibit most citizens from using it)
Public information campaigns	0—No COVID-19 public information campaign 1—Public officials urging caution about COVID-19 2—Coordinated public information campaign (e.g., across traditional and social media)
Stay at home	0—No measures 1—Recommend not leaving house 2—Require not leaving house with exceptions for daily exercise, grocery shopping, and “essential” trips 3—Require not leaving house with minimal exceptions (e.g., allowed to leave only once every few days, or only one person can leave at a time, etc.)
Restrictions on internal movement	0—No measures 1—Recommend movement restriction 2—Restrict movement
International travel controls	0—No measures 1—Screening 2—Quarantine arrivals from high-risk regions 3—Ban on high-risk regions 4—Total border closure
Testing policy	0—No testing policy 1—Only those who both (a) have symptoms AND (b) meet specific criteria (e.g., key workers, admitted to hospital, came into contact with a known case, or returned from overseas) 2—Testing of anyone showing COVID-19 symptoms 3—Open public testing (e.g., “drive through” testing available to asymptomatic people)
Contract tracing	0—No contact tracing 1—Limited contact tracing—not done for all cases 2—Comprehensive contact tracing—done for all cases
Face coverings	0—No policy 1—Recommended 2—Required in some specified shared/public spaces outside the home with other people present, or some situations when social distancing not possible 3—Required in all shared/public spaces outside the home with other people present or all situations when social distancing not possible 4—Required outside the home at all times regardless of location or presence of other people
Vaccination policy	0—No availability 1—Availability for ONE of following: key workers/clinically vulnerable groups/elderly groups 2—Availability for TWO of following: key workers/clinically vulnerable groups/elderly groups 3—Availability for ALL of following: key workers/clinically vulnerable groups/elderly groups 4—Availability for all three plus partial additional availability (select broad groups/ages) 5—Universal availability

**Table 3:** Classification of policy stringency.

Policy Stringency	Levels of Policies for Management and Control	Conditions of Policies for Management and Control
0–25	The first level	Looser control
25–50	The second level	Loose control
50–75	The third level	Strict control
75–100	The fourth level	Stricter control

**Table 4:** Continuity test for control variables in regression discontinuity.

Stringency	Tdeaths_pm	Coef.	z	P > |z|	95% Conf.	Interval
25	m_age	−6.8792	−3.5700	0.0000	−10.6537	−3.1047
	gdp_pc	6270.8690	1.2200	0.2210	−3771.3140	16,313.0500
	e_poverty	19.3998	3.8400	0.0000	9.4890	29.3107
	f_smokers	1.7810	0.7100	0.4770	−3.1310	6.6931
	m_smokers	0.2632	0.1000	0.9230	−5.0688	5.5953
	handwash	−63.8128	−6.6100	0.0000	−82.7468	−44.8788
	hbp	0.4461	0.9300	0.3510	−0.4908	1.3831
50	m_age	−1.2390	−0.8200	0.4100	−4.1849	1.7069
	gdp_pc	5272.5470	1.9800	0.0470	66.4699	10,478.6200
	e_poverty	9.9797	2.0900	0.0360	0.6305	19.3289
	f_smokers	−0.0423	−0.0300	0.9750	−2.7144	2.6298
	m_smokers	5.4180	2.9500	0.0030	1.8129	9.0230
	handwash	7.9060	1.5900	0.1120	−1.8543	17.6662
	hbp	0.4962	1.0300	0.3020	−0.4467	1.4391
75	m_age	−5.0188	−7.3600	0.0000	−6.3558	−3.6818
	gdp_pc	−9404.2130	−6.2100	0.0000	−12,370.3100	−6438.1190
	e_poverty	4.6437	3.7900	0.0000	2.2431	7.0443
	f_smokers	−1.8714	−2.0600	0.0390	−3.6483	−0.0944
	m_smokers	0.0253	0.0200	0.9830	−2.2657	2.3163
	handwash	−24.8325	−7.2100	0.0000	−31.5844	−18.0807
	hbp	0.0582	0.3100	0.7590	−0.3144	0.4309

**Table 5:** Fuzzy regression discontinuity of stringency and tdeaths_pm.

Stringency	Tdeaths_pm	Coef.	Std. Err.	z	P > |z|	95% Conf.	Interval
25	numer	−0.2409	0.1118	−2.1500	0.0310	−0.4601	−0.0217
	denom	0.6479	0.0634	10.2200	0.0000	0.5236	0.7721
	lwald	−0.3718	0.1749	−2.1300	0.0340	−0.7147	−0.0289
50	numer	−33.8400	7.5731	−4.4700	0.0000	−48.6830	−18.9970
	denom	0.9834	0.0116	84.6900	0.0000	0.9606	1.0062
	lwald	−34.4109	6.2777	−5.4800	0.0000	−46.7149	−22.1069
75	numer	−29.6531	6.3416	−4.6800	0.0000	−42.0824	−17.2238
	denom	0.8805	0.0176	50.0300	0.0000	0.8460	0.9149
	lwald	−33.6793	7.2702	−4.6300	0.0000	−47.9287	−19.4299

**Table 6:** Bandwidth dependence test of regression discontinuity.

Type	Stringency	Tdeaths_pm	Coef.	Std. Err.	z	P > |z|	95% Conf.	Interval
fuzzy	25	lwald	−0.3718	0.1749	−2.1300	0.0340	−0.7147	−0.0289
		lwald50	−1.1889	1.2968	−0.9200	0.3590	−3.7305	1.3527
		lwald200	−0.2783	0.1886	−1.4800	0.1400	−0.6480	0.0914
	50	lwald	−34.4109	6.2777	−5.4800	0.0000	−46.7149	−22.1069
		lwald50	−16.1610	4.4426	−3.6400	0.0000	−24.8683	−7.4536
		lwald200	−36.6040	5.2472	−6.9800	0.0000	−46.8882	−26.3197
	75	lwald	−33.6793	7.2702	−4.6300	0.0000	−47.9287	−19.4299
		lwald50	4.7146	7.7384	0.6100	0.5420	−10.4523	19.8816
		lwald200	−28.0387	5.3107	−5.2800	0.0000	−38.4476	−17.6299

## Data Availability

Data available in a publicly accessible repository. The data presented in this study are openly available in https://ourworldindata.org.
